# Optimal economic productivity of exopolysaccharides from lactic acid bacteria with production possibility curves

**DOI:** 10.1002/fsn3.1079

**Published:** 2019-06-03

**Authors:** Tachen Lin, Chunyeh Chen, Bangyuan Chen, Jeifu Shaw, Yuhshuen Chen

**Affiliations:** ^1^ College of Food Engineering Beibu Gulf University Qinzhou China; ^2^ Guangxi Colleges and University Key Laboratory of Development and High‐value Utilization of Beibu Gulf Seafood Resources Beibu Gulf University Qinzhou China; ^3^ Department of Food Science and Technology Hungkuang University Shalu District, Taichung City Taiwan; ^4^ Department of Food Science Fu Jen Catholic University Xinzhuang District, New Taipei City Taiwan; ^5^ Department of Biological Science and Technology I‐Shou University Dashu District, Kaohsiung City Taiwan

**Keywords:** antioxidant ability, exopolysaccharides, lactic acid bacteria

## Abstract

It is important that exopolysaccharides (EPS) of lactic acid bacteria (LAB) with antioxidant activities are produced economically, as it can confer beneficial effects on human health. A model of production possibility curve (PPC) was conducted to the optimal productivity of EPS for the purpose of economic production. The results revealed that the optimal productivity of EPS was approached to the set by NB (90%) and MRS (55%) broth from PPC with equation of PPC(Y) = 100–0.0335*EXP(0.08*X). The EPS productivity and yield of strain LaP with optimal production set (OPS) were, respectively, 291.0 ± 2.6 mg and 13.5 ± 0.7%, and the cost of OPS can be saved by about 31.6%, while that for strain BaP were 280.7 ± 2.5 mg and 13.0 ± 0.7%, respectively, and with 31.6% saved as well. Besides, the EPS produced from PPC mode has appropriate antioxidant ability with 34.6 ± 0.7% (LaP) and 37.6 ± 0.9 (%) (BaP) of DPPH radical scavenging activity under the economic cost. The strategy of controlling the medium composition not only could improve the productivity of EPS, but also enhance the antioxidant effects of EPS. Both LaP and BaP with antioxidant potential may be useful as supplements in the health‐promoting food industry.

## 1 INTRODUCTION

The exopolysaccharides (EPS) fermented by lactic acid bacteria (LAB) has been widely used in industrial applications and good health benefits in daily life, which has gained the focus of considerable research, and some are mainly focused on health function and production (Huang, Huang, Kao, & Fang, 2017; Inturri et al., 2017), but few productivity and production cost were of concerns. However, the industrial applications of EPS are hindered by the low yield of EPS from LAB and high costs of their purification (Oleksy & Klewic, 2018). This is because the EPS yield is a key limiting factor in the utilization of EPS, and so the strategies for enhancement of EPS production are considerable researched (Jiang, Hao, Qu, & Hu, 2017). The EPS production in literature has been widely explored by using the theoretical methods of mathematics, physics, chemistry, and biology (Hsu, Wu, Lin, Lum, & Cheng, 2017; Jiang & Yang, 2018; Kho et al., 2016; Moghannem, Farag, Shehab, & Azab, 2018; Oldak, Zielinska, Rzepkowska, & Kolozyn‐Krajewska, 2017; Soanen, Silva, Gardarin, Michaud, & Laroche, 2016), but the application of EPS production by using is less the methodology of economic theory (Badel, Bernardi, & Michaud, 2011). Due to the cost of production, it is reasonable to discuss the EPS productivity based on economic theory. Moreover, it is worthy of study that the productivity and cost are closely related to expand application of industrial production and scale up of economic problems. The innovation of this study is using application of economic theory for microbial fermentation to develop the production mode of optimal economic medium and reduce production costs. Therefore, the strategies for promoting productivity and cost reduction of EPS could include chemical media of formula enhancement and physical production of cost reduction. Thus, the experimental designs were achieved in two ways: (a) formula adjustment (b) Pareto optimality theory (Ranganathan, Fafchamps, & Walker, 1991). The detail experiments were performed as follows: (a) productivity enhanced by formula adjustment, including medium adjustment with using some Man–Rogosa–Sharpe (MRS) broth by individual addition of nutrient broth (NB), glucose, lactose, fructose, and E broth. These were conducted to develop the PPC model. (b) Cost reduction of EPS production: It would be achieved by amounts of reduction of medium composition and development of PPC model to obtain the optimal production set of EPS production.

Concerning formula adjustment strategies for enhancement of EPS production by LAB, the production of a desired EPS could be achieved by controlling the culture conditions (Ruas‐Madiedo, Hugenholtz, & Zoon, 2002). In some instances, EPS structure has been found to depend on the carbon source. Structural analyses of the EPS produced by *L. delbrueckii* subsp. *bulgaricus* NCFB 2772 grown in continuous culture showed that the EPS consisted of repeating units of glucose and galactose (in the ratio of 1:2.4) when grown on fructose (German et al., 1999; Jiang et al., 2017). The other case is that the EPS consisted of glucose, galactose, and rhamnose in a ratio of 1:7.0:0.8 when grown on a mixture of fructose and glucose (German et al., 1999). Thus, the understanding of EPS structure will improve the EPS biosynthesis, increase the productivity of EPSs, and develop EPSs with desirable properties (Ispirli & Dertli, 2017; Jiang et al., 2017).

**Figure 1:**
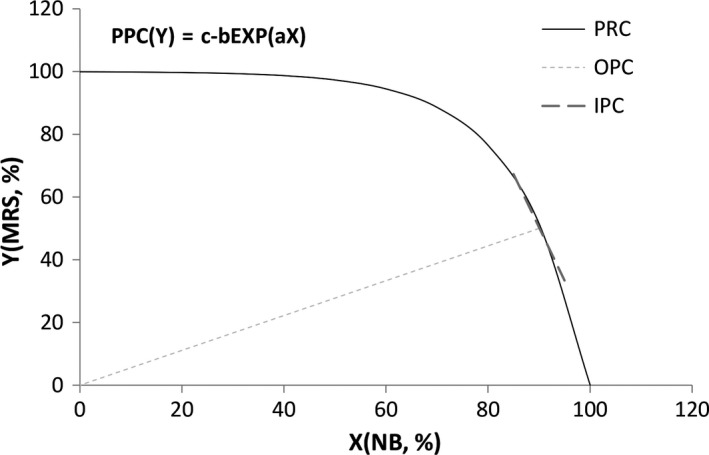
Schematic diagram of production possibility curve with the optimal productivity set of media MRS broth and NB. IPC, isotherm production curve; OPC, optimal productivity cure; PPC, production possibility curve

For the purpose of economic production, the POT and PPC of economics were conducted (Ranganathan et al., 1991). From the view point of economics, the most economic production set can be approached according to Pareto optimality theory (POT) (Ranganathan et al., 1991). The condition of POT could be interpreted by the production efficiency (MRSxy) and transaction efficiency (MRTxy). The optimal productivity set (OPS) is determined by the PPC in which each point has the good production efficiency. The tangency of the commodity price curve (Px/Py) to the PPC is the OPS as shown in Figure 1, that is, MRSxy = MRTxy = Px/Py. Hence, the tangent point on the PPC is the optimal productivity.

The study proposes to improve the productivity of EPS and to reduce cost of EPS production. The strategies were performed as follows: (a) Strategies for enhancing the EPS yields: The strategies included the formula adjustment enhanced with various carbon sources. (b) Cost reduction of EPS production: The strategies included amounts of reduction of medium compositions and development of PPC model to obtain the OPS of EPS production. Besides, 2,2‐Diphenyl‐1‐picrylhydrazyl (DPPH) radical scavenging activity of EPS from OPS was used to assess the antioxidant function for health food.

## 2 MATERIALS AND METHODS

### 2.1 Strains and medium

Lactic acid bacteria of EPS production were screened from fermented yogurt, and two strains were isolated and then identified as *Lactobacillus acidophilus* (La) and *Bifidobacterium adolescentis* (Ba). Both strains were cultured on MRS medium (Difco) with some modification for improving the high productivity and yield of EPS. The MRS broth was used to incubate strains La and Ba to harvest EPS as a control group. The MRS broth in addition to various components including 5 g/L NB, 5 g/L glucose, 5 g/L lactose, 5 g/L fructose or E medium was used as experimental groups, respectively. The E medium was defined as the optimal chemical medium with C, N, P, and metal ion source in a ratio of 100%:10%:1.0%:0.1% through series concentration of optimal growth test.

### 2.2 Cultivation and inoculation

About 1% (v/v) LAB were inoculated into 100 or 300 ml MRS broth as control group and 100 or 300 ml broth of various media as experimental groups and cultured at 40°C, pH 6.0 for 48 hr under anaerobic conditions. The production and yield of EPS were estimated for comparison between control group of MRS medium and experimental groups of various media.

### 2.3 Exopolysaccharides preparation

The preparation of EPS produced by strains La and Ba was carried out according to the method by Liu et al. (2011) with some modifications. In brief, strains Ba and La were cultured at 40°C for 48 hr, respectively. After centrifugation at 5,000 *g* for 20 min, the precipitate was collected to dry in 105°C oven for 48 hr, then weighed as dry cell weight. The supernatant was added by 0.4 mol/L trichloroacetic acid (Baker) and stood at 4°C for 3 hr. The extracts were pooled, concentrated to 30% of the original volume under a reduced pressure using Ratavapor R210 and then centrifuged at 5,000 *g* for 20 min. The supernatants were collected and were added with 95% alcohol with 3 volume of supernatant by slowly stirring to precipitate the polysaccharides (Cooper & Goldenberg, 1987) and then kept at 4°C for 24 hr, followed by centrifugation at 5,000 *g* at 4°C for 20 min. Then, the precipitate was dialyzed using Snake Skin@ dialysis tubing (MWCO 10,000 Da, Thermo Scientific) for 24 hr. The EPS pellets were obtained by centrifugation at 5,000 *g* for 20 min and repeatedly washed with some ethanol. Finally, the exopolysaccharide pellets were lyophilized in Utek Twcess freeze dryer (Lyophilization System) for 48 hr. The freeze‐dried powders of BaP and LaP, respectively, produced by strains Ba and La were weighed and stored at −20°C before use (Liu et al., 2011; Tseng et al., 2012). The yield (%) of EPS can be calculated by the weight of EPS produced divided by dry cell weight and expressed as percentage.

### 2.4 Enhancement of EPS production with formula adjustment

The promotion of EPS production is achieved by using formula adjustments, enhanced with various carbon sources. The suggested concentration of MRS broth was first adjusted from 55 to 50 g/L and was enhanced by individually supplied with 5 g of various carbon sources such as glucose, lactose, and fructose. One milliliter starters of 1% strains La and Ba was individually inoculated into 100 ml MRS broth of formula adjusted in 250‐ml flasks and cultured at 40°C for 48 hr under anaerobic conditions. The dry cell weight and EPS produced were measured to estimate the production and yield of EPS. The yield of EPS was calculated as the percentage of the EPS weight divided by the dry cell weight. All the experiments were performed in triplicates.

### 2.5 Cost reduction of EPS production by amounts reduction of medium composition

Cost reduction of EPS production included reducing formula amount and developing a model of PPC. The reduction of formula amount was carried out as follows: the formula amount of MRS broth was remarkably reduced to half amount of formula direction for economic production. Thus, the MRS broth of a traditional formula was adjusted from about 55 g/L of suggested usage to 20 g/L and was supplied with 5 g/L NB and 5 g/L (about 10% amount of MRS broth) of various sugar compositions such as glucose, lactose, or fructose. Then, the total amount of broth was about 30 g/L and was over half of 55 g/L. Therefore, considering the economic productivity and yields of EPS, the experimental design was adapted by combination of 20 g/L MRS broth and 5 g/L NB broth with various nutrient compositions, which individually included 20% glucose (Glu), 20% lactose (Lac), or 20% fructose (Fru).

### 2.6 Model development of production possibility curve

According to the model development for the PPC, the two factors of commodity price curve (Px/Py) could be constituted by the consumption percentage of media MRS (Y) and NB (X) as shown in Figure 1, owing that MRS is generally used for LAB cultivation and NB is general for microbiology cultivation. The consumption percentages of MRS and NB media were presented as two producing factors for EPS production by strains La and Ba. More expensive MRS broth (200 USD/kg) could be partly replaced by cheaper NB (120 USD/kg) for the purpose of economic production in commercial consideration. In ideal conditions, the consumption percentage of NB (X) and MRS (Y) media could satisfy the PPC according to POT (Ranganathan et al., 1991), and then, the optimal productivity set of EPS could be approached. The slope of isotherm production curve (IPC) will be tangent to PPC, when the production efficiency (MRSxy) and transaction efficiency (MRTxy) are equal. Thus, the OPS will be gained by the IPC, and the equation of optimal productivity cure (OPC) could be obtained by the tangent point of the IPC as shown in Figure 1. Hence, the economic production of EPS was dependent on the consumption percentage of NB (X) and MRS (Y) media. Moreover, the difference in consumption percentage of MRS (Y) media will be gradually increasing, depending on the decreasing difference in consumption percentage of NB (X) on the OPC.

### 2.7 Economic production by optimal productivity sets

According to the POT, seven experimental groups of optimal productivity sets combined by media MRS and NB were performed to produce EPS. Therefore, the production, yield, and producing cost per liter were in comparison. The purpose of the experimental design was to develop a lower cost medium for the EPS production, which the lower prices of NB could be partly substituted for the higher prices of MRS broth. Therefore, the economic production could be approached by optimal productivity set.

### 2.8 DPPH radical scavenging activity assay

The radical scavenging activity of the fermented solution of each set from experimental PPC against stable DPPH was determined using a slightly modified DPPH radical scavenging assay (Chen, Lin, & Chen, 2015). Briefly, a 25 ml fermented solution was diluted to 4 ml by using methanol and then mixed with 0.5 ml of a freshly prepared 1 mM DPPH solution (Sigma Chemical Co.). After 30 min of incubation, the absorbance at 517 nm was measured using a spectrophotometer (U‐2001, Hitachi Co.). The DPPH radical scavenging activity was calculated by using the following equation (Chen et al., 2015):

$$\text{DPPH radical scavenging activity}\left(\%\right)=\left[1-\left({\text{OD}}_{517}\;\text{of sample with fermented solution}\right)/\left({\text{OD}}_{517}\;\text{of control without fermented solution}\right)\right]\times 100.$$

### 2.9 Statistics analysis

Each experiment was repeated three times and analyzed via SAS software (version 8.0 235 Ed.). Duncan's multiple range test was used to analyze the difference among the treatments.

## 3 RESULTS

### 3.1 Enhancement of EPS production by formula adjustment

The productivity and yields of EPS for strain La cultivated in MRS broth were, respectively, 303.3 ± 3.5 mg/L and 11.6 ± 1.0%; while in strain Ba were, respectively, 291.7 ± 1.5 mg/L and 11.5 ± 0.6% (Table 1). Thus, the productivity and yields of EPS for strain La were superior to that of strain Ba in MRS broth. The productivity and yields of EPS for strains La and Ba incubated in MRS broth with various carbon sources, such as glucose, lactose, and fructose, were significantly higher than that in MRS broth only (*p* < 0.05). The optimal productivity and yields of EPS for strains La were, respectively, 491.0 ± 3.6 mg/L and 12.4 ± 0.6%, which was incubated in MRS broth with 5 g lactose, while those in strain Ba were, respectively, 498.7 ± 6.4 mg/L and 12.3 ± 0.9%, which was incubated in MRS broth with 5 g glucose. The optimal EPS production of both strains La and Ba was significantly higher than the other experiments as shown in Table 1 (*p* < 0.05).

**Table 1:** The EPS yields produced by 1% (v/v) of strains La and Ba incubated in 100 ml MRS broth with additive of 5 g glucose or lactose or fructose at 40°C for 48 hr under anaerobic conditions

Strains	Medium (55 g/L)	Dry cell weight (g/L)	Exopolysaccharide produced (mg/L)	Yield (%)
La	MRS broth	2.6 ± 0.3	303.3 ± 3.5^a^	11.6 ± 1.0
	MRS broth + 5 g glucose	3.2 ± 0.4	387.7 ± 2.5^c^	12.2 ± 1.5
	MRS broth + 5 g lactose	4.0 ± 0.2	491.0 ± 3.6^d^	12.4 ± 0.6
	MRS broth + 5 g fructose	3.0 ± 0.3	358.3 ± 3.5^b^	12.0 ± 1.0
Ba	MRS broth	2.5 ± 0.2	291.7 ± 1.5^a^	11.5 ± 0.6
	MRS broth + 5 g glucose	4.1 ± 0.4	498.7 ± 6.4^d^	12.3 ± 0.9
	MRS broth + 5 g lactose	3.3 ± 0.2	395.3 ± 5.5^c^	12.1 ± 0.7
	MRS broth + 5 g fructose	3.1 ± 0.3	368.3 ± 3.5^b^	11.8 ± 1.1

Note

The characters of a, b, c, and d in the column of exopolysaccharide produced are different at a significance level of *p* < 0.05.

Abbreviations: Ba, *Bifidobacterium adolescentis*; EPS, exopolysaccharide; La, *Lactobacillus acidophilus*.

### 3.2 Cost down of EPS production by amounts reduction of medium composition

Considering the economic productivity and yields of EPS, the experimental design was adapted by combination of 20 g/L MRS broth and 5 g/L NB broth with various nutrient compositions, which individually included about 20% glucose (Glu), 20% lactose (Lac), or 20% fructose (Fru) of total amounts of 20 g/L MRS broth and 5 g/L NB broth. In comparison of MRS broth and MRS/NB with various nutrient compositions, the results revealed that the productivity and EPS yields of strain La and Ba incubated in MRS/NB with various sugars were superior to that of strain La and Ba in MRS broth only (Table 2). The highest productivity and EPS yield of LaP were, respectively, 397.3 ± 2.1 mg/L and 12.6 ± 0.6%, which were incubated in 20 g/L of MRS broth and 5 g/L NB broth with 5 g/L lactose (Lac) except for in MRS/NB + E broth. As for BaP, the highest productivity and EPS yield were, respectively, 412.0 ± 5.3 mg/L and 12.6 ± 0.7%, which were incubated in 20 g/L of MRS broth and 5 g/L NB broth with 5 g/L glucose (Glu). The optimal EPS production of both strains La and Ba was significantly higher than the other experiments as shown in Table 2 (*p* < 0.05).

**Table 2:** The EPS yields produced by *Lactobacillus acidophilus* (La) and *Bifidobacterium adolescentis* (Ba) incubated in different MRS broth with various nutrient compositions at 40°C for 48 hr under anaerobic conditions

Medium	La		Ba	
	Exopolysaccharide produced (mg/L)	Yield (%)	Exopolysaccharide produced (mg/L)	Yield (%)
MRS broth	298.7 ± 3.2^d^	11.8 ± 0.6	285.7 ± 4.0^d^	11.5 ± 1.5
MRS/NB + E broth	388.4 ± 6.2^e^	15.5 ± 0.8	342.3 ± 2.3^e^	14.3 ± 0.7
MRS/NB + 5 g/L glucose	343.7 ± 1.5^a^	12.3 ± 0.5	412.0 ± 5.3^a^	12.6 ± 0.7
MRS/NB + 5 g/L lactose	397.3 ± 2.1^b^	12.6 ± 0.6	356.7 ± 7.6^b^	12.3 ± 0.4
MRS/NB + 5 g/L fructose	319.7 ± 1.5^c^	12.0 ± 0.7	315.0 ± 3.0^c^	12.0 ± 0.7

Note

The experimental groups MRS/NB combined 20 g/L of MRS broth and 5 g/L NB broth with 5 g/L various nutrient compositions containing glucose, lactose, or fructose, while the control group was 55 g/L of MRS broth. The characters of a, b, c, and d in the column of exopolysaccharides produced are different at a significance level of *p* < 0.05. E broth, the optimal chemical medium with C, N, P, and metal ion source in a ratio of 100%:10%:1.0%:0.1% through series concentration of optimal growth test.

Abbreviations: Ba, *Bifidobacterium adolescentis*; La, *Lactobacillus acidophilus*; NB, nutrient broth.

### 3.3 Model development of production possibility curve

**Figure 2:**
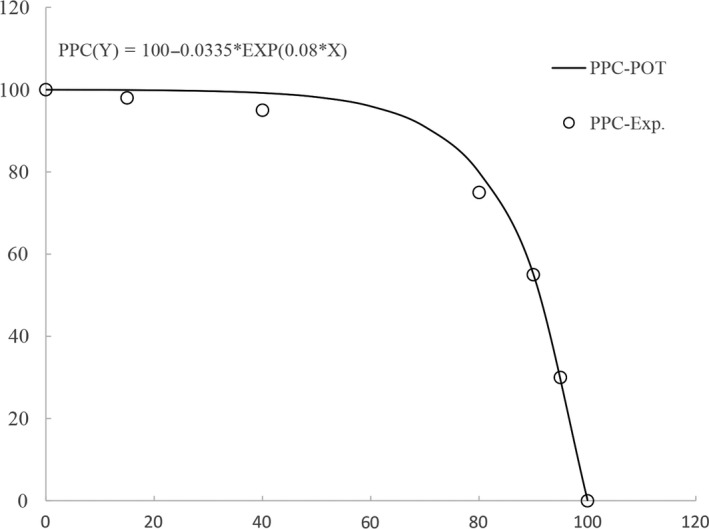
The optimal productivity set from model of product possibility curve with two medium NB (90%) & MRS (55%). IPC, isotherm production curve; OPC, optimal productivity cure; PPC, production possibility curve

The PPC was conducted and fitted to the model by seven sets of batch experiment as shown in Figure 2 with the equation of PPC(Y) = 100–0.0335EXP(0.08X), *R* ^2^ = 0.9696, IPC(Y) = −0.619(X) + 55.7, and OPC(Y) = 0.611(X). Therefore, the optimal productivity set (OPS) can be obtained from the tangent point of the isotherm production curve (IPC) to the PPC model equation and was NB (90%) and MRS (55%), which completely fitted the set of NB (90%) and MRS (55%) from the PPC. The results also revealed that the EPS yield was increased with the NB amount and the EPS yield was the maximum when it reached to the first best point, that is, OPS (90%, 55%). Moreover, for the optimal productivity, there was over 3.5 USD/kg reduction in cost of the highest yield of OPS batch experiment per liter in comparison with that of MRS broth only.

### 3.4 Economic production by optimal productivity set

According to Pareto optimality model of PPC with equation of PPC(Y) = 100–0.0335*EXP(0.08*X), the results of EPS production and yield of PPC from strain LaP and BaP were shown in Tables 3 and 4, respectively. The optimality of EPS production of both strains LaP and BaP was 291.0 ± 2.6 and 280.7 ± 2.5 mg/L, respectively. They all occurred in the optimal productivity set of NB (90%) and MRS (55%), and the yield of both strains LaP and BaP was, respectively, 13.5 ± 0.7% and 13.0 ± 0.7% and was the maximum when compared to the other experimental set. The optimal cost of batch experiments is about 7.50 USD/L in both strains La and Ba. However, the cost of OPS batch experiment per liter with the optimal productivity set was significantly lower than other sets of batch experiments, except for that with the amount of MRS <50% (*p* < 0.05).

**Table 3:** The EPS production and yield of product possibility curve from strain La with equation of PPC(Y) = 100–0.0335*EXP(0.08*X)

NB (X, %)	MRS (Y, %)	Dry cell weight (g)	EPS production (mg)	Yield (%)	OPS cost of batch experiment (USD/L)
0	100	2.6 ± 0.3	307.0 ± 5.0	11.7 ± 1.2	11.00^c^
15	98	2.4 ± 0.2	300.7 ± 4.5	12.4 ± 0.9	11.01^c^
40	95	2.3 ± 0.2	301.3 ± 4.0	13.2 ± 1.0	11.05^c^
80	75	2.2 ± 0.2	287.7 ± 5.1	13.3 ± 0.7	8.15^b^
90	55	2.2 ± 0.1	291.0 ± 2.6	13.5 ± 0.7	7.40^a^
95	25	1.5 ± 0.1	194.7 ± 4.5	13.0 ± 0.8	4.18
100	0	1.1 ± 0.1	107.0 ± 5.0	9.5 ± 0.7	1.50

Note

The characters of a, b, c, and d in the OPS cost are different at a significance level of *p* < 0.05.

Abbreviations: EPS, exopolysaccharide; La, *Lactobacillus acidophilus*; NB, nutrient broth; OPS, optimal productivity set.

**Table 4:** The EPS production and yield of production possibility curve from strain Ba with equation of PPC(Y) = 100–0.0335*EXP(0.08*X)

NB (X, %)	MRS (Y, %)	Dry cell weight (g/L)	EPS production (mg/L)	Yield (%)	OPS cost of batch experiment (USD/L)
0	100	2.7 ± 0.2	305.7 ± 2.1	11.5 ± 0.7	11.00^c^
15	98	2.5 ± 0.2	300.3 ± 4.0	12.1 ± 0.9	11.01^c^
40	95	2.4 ± 0.2	295.0 ± 6.6	12.5 ± 1.0	11.05^c^
80	75	2.2 ± 0.2	282.0 ± 1.0	12.7 ± 0.8	8.15^b^
90	55	2.2 ± 0.1	280.7 ± 2.5	13.0 ± 0.7	7.40^a^
95	25	1.4 ± 0.1	175.7 ± 2.5	12.6 ± 1.0	4.18
100	0	1.1 ± 0.1	102.7 ± 2.1	9.1 ± 0.9	1.50

Note

The characters of a, b, c, and d in the OPS cost are different at a significance level of *p* < 0.05.

Abbreviations: Ba, *Bifidobacterium adolescentis*; EPS, exopolysaccharide; NB, nutrient broth; OPS, optimal productivity set.

### 3.5 OPS of production possibility curve

The costs of OPS from PPC were about 7.5 USD/L and were about two‐third of the cost of MRS broth in both strains La and Ba as shown in Tables 5 and 6. There were over 3.0 USD/L reductions (LaP, 3.49 USD/L [31.6%]; BaP, 3.48 USD/L [31.6%]) in cost of each batch experiment of OPS in comparison with that of non‐PPC. Moreover, the results further revealed that the cost of OPS from PPC can be saved more by 30% at least in comparison with that of MRS broth only.

**Table 5:** LaP production cost of the optimal productivity set with E medium

Ea broth^a^ composition	Dosage used (%)	Dosage used (g)	Unit price (USD)	OPS cost^b^ (USD/L)	MRS broth cost (USD/L)
MRS	55	28	100	5.67	11.00
NB	90	14	67	1.8	
Lactose	20	8.4	5.7	0.1	
NH_4_Cl	3	1.26	3.2	0.01	
K_2_HPO_4_	0.2	0.084	5.2	0.001	
CaSO_4_	0.04	0.0168	3.2	0.0001	
				7.51	3.49 (31.7%)

Note

The total cost of OPS is 7.51 USD/L which is lower than that with 55 g per liter MRS broth by 3.49 USD/L.

Abbreviation: LaP, exopolysaccharide produced by strain La; OPS, optimal productivity set.

a Ea broth, E medium that strain La growth prefers lactose‐base medium.

b Unit price from local suppliers.

**Table 6:** BaP production cost of the optimal productivity set with E medium

Eb broth^a^ composition	Dosage used (%)	Dosage used (g)	Unit price (USD)	OPS cost^b^ (USD/L)	MRS broth cost (USD/L)
MRS	55	28	100.00	5.67	11.00
NB	90	14	66.67	1.80	
Glucose	20	8.4	3.00	0.05	
NH_4_NO_3_	3	0.42	3.20	0.001	
KH_2_PO_4_	0.2	0.21	6.00	0.002	
MgSO_4_	0.04	0.0168	2.80	0.001	
				7.52	3.48 (31.6%)

Note

The total cost of OPS is 7.51 USD/L which is lower than that with 55 g per liter MRS broth by 3.48 USD/L.

Abbreviation: BaP, exopolysaccharide produced by strain Ba; OPS, optimal productivity set.

a Eb broth, E medium that strain Ba growth prefers glucose‐base medium.

b Unit price from local suppliers.

### 3.6 2,2‐Diphenyl‐1‐picrylhydrazyl radical scavenging activity

**Figure 3:**
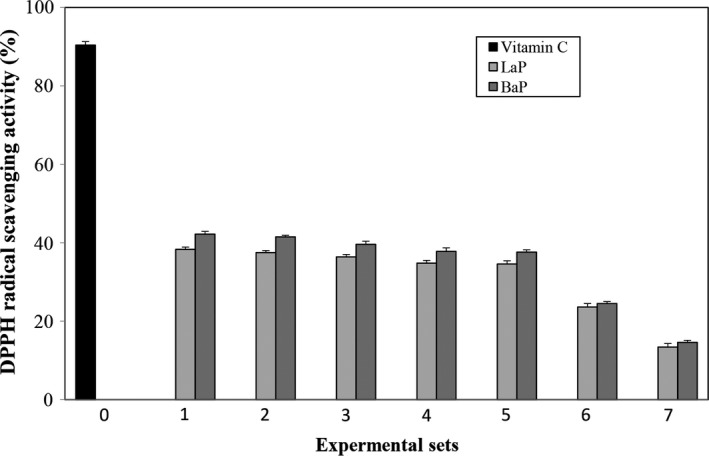
DPPH radical scavenging activity of experimental sets from PPC mode. 0: Vitamin C was used as the positive control. Sets 1–7: (0,100); (15,98); (45,95); (80,75); (90,55); (95,25); (100,0) in percent of NB & MRS medium combination. The characters of a, b, c, and d in the figure are significantly different from experiment sets (*p* < 0.05). Each set is triple experiments (*n* = 3)

DPPH radical scavenging activity exhibited an important antioxidative ability. The results shown in Figure 3 indicate that there were statistically significant differences in four experimental groups (*p* < 0.05). The DPPH radical scavenging activity of the first three groups (a: 1–3) of experiments (0, 100; 15, 98; 45, 95) was not statistically significant differences (*p* > 0.05). The latter two groups (b: 4, 5) were statistically significantly different from the first three groups, but they also significantly had 90% activity of the first three groups (*p* < 0.05), while those in the third group (c: 6) were only 2/3 of the first three groups. Therefore, DPPH radical scavenging activity responded to the yield of EPS from each experimental set. Thus, EPS produced from PPC mode has appropriate antioxidant function and yield under the economic cost.

## 4 DISCUSSION

As for enhancement of EPS production by formula adjustment, the effects of strategies for EPS yields using formula adjustment enhanced with various carbon sources were different as shown in Tables 1 and 2. The purpose of 5 g/L NB addition is to supply with enough nutrients and mineral metals. Meanwhile, the additive purpose of 5 g/L various carbon sources may play a role to supply with an inducer or primary nutrient for growth (Castellane et al., 2017; Rios‐Covian et al., 2015). The productivity and yields of EPS for strain La was enhanced by lactose, while strain Ba was enhanced by glucose. Thus, the results showed that monosaccharide or disaccharide such as glucose and lactose have easy digestion to enhance the bioreaction of growth of LAB. Hence, the role of 5 g/L glucose, lactose, or fructose was an inducer for growth of strains La and Ba. The inducer with small molecule of various carbon sources could induce the strain growth then promote the EPS production and yield. Moreover, the addition of fermentable sugars (glucose, lactose, and fructose) can be used as an electron acceptor by LAB and resulted in acetate formation (Gänzle, Vermeulen, & Vogel, 2007). An excess of acetate can stimulate the LAB growth and improve monosaccharide unit of EPS synthesis (Galle, Schwab, Arendt, & Gänzle, 2010; Galle et al., 2012). Gopal, Prasad, Smart, and Gill (2001) also demonstrated that genera *Bifidobacterium* and *Lactobacillus* such as *B. lactis* DR10 and *Lb. rhamnosus* DR20 preferentially utilized sugars with a low degree of polymerization, that is disaccharides, monosaccharides, tri‐, and tetra‐saccharides which were likely to promote growth of LAB. Therefore, the effect of sugars such as glucose, galactose, lactose, and fructose on exopolysaccharides production could play an important role of precursor on biosynthesis (Audy, Labrie, Roy, & LaPointe, 2010). EPS production started in the early growth phase and stopped in the early stationary phase (Van den Berg et al., 1995). Therefore, the EPS production will increase with the amount of cell growth. In general, the production of EPS by different LAB varies roughly from 50 to 350 mg/L under nonoptimized culture conditions (Cerning, 1990; van den Berg et al., 1995). The productivity and EPS yield of strains La and Ba cultivated in three different experimental media were, respectively, 1.1–1.3, 1.0–1.1 times and 1.0–1.4, 1.0–1.2 times in LaP and BaP higher than that in the control group of MRS broth (Table 2). Moreover, strain La seems to prefer lactose‐base medium while strain Ba grew well in the glucose‐base medium (Table 2). It was also revealed that both strains may involve different metabolic pathways of EPS production (Liu et al., 2011; Welman & Maddox, 2003).

Concerning cost reduction of EPS production by amounts reduction of medium composition, one of the strategies for cost reduction of EPS production can be achieved by amount reduction of medium compositions. Generally, common culture medium for LAB is MRS. The local commercial price of MRS broth per bottle of 500 g is about USD $ 83–116 (Backer or Sigma), and its indication of usage amount is about 55 g/L. On the contrast, the local commercial price of widely used NB is about USD $ 50–67 per bottle of 500 g, and its indication of usage amount is about 15 g/L. Thus, not only is the commercial price of MRS broth more expensive than that of NB by 1.6–1.75 times, but also the consumption of MRS broth is higher than that of NB broth by 3.7 times. Hence, there are 5.9–6.5 times of difference in the total cost. In order to save costs for achieving economic production, the usage amount of MRS broth with higher cost shall be reduced and replaced by lower cost of NB. The strategy for economic production of the EPS is to reduce the usage amounts from 55 to 20 g/L and addition of 5 g/L (ca. 20%) NB broth for nutrient supplement. Thus, the EPS production per unit cost can be saved by about USD $ 6.3 per liter. Hence, the adjusted strategy for the purposes of economic production can be achieved by 57.3% of cost reduction.

As for OPS of production possibility curve, the dry cell weight of LAB decreased with the decrease of MRS concentration, while the yield of EPS increased with the increase of NB concentration, indicating that 55% amount of MRS was sufficient to support the growth of LAB, while NB could be used to promote the production of EPS (Tables 3 and 4). The producing amount of EPS depended on the substrate of LAB growth and high EPS production correlated with high protein, fermentable sugars such as glucose, lactose, fructose, and mineral contents in substrate (Wolter et al., 2014). This indicated why 90% amount of NB replacing 45% MRS broth with high amount nutrition in substrate succeeds to enhance higher EPS production. The production costs can be reduced by more than 1/3, about 37.90%, and the productivity can approach to nearly 95% (94.7%). Thus, economic production of EPS from *Lactobacillus acidophilus* La and *Bifidobacterium adolescentis* Ba could be approached by using the optimal productivity set of PPC model. This is consistent with Pareto's optimization efficiency, that is, to maximize the economic efficiency of the production at the least cost.

Concerning antioxidant activity**,** biological activity and stability could be estimated by considering the ability to eliminate DPPH free radical oxidation. The DPPH radical scavenging activity was based on determining the reduced concentration of a DPPH solution by using an antioxidant. The 55% of MRS amount was about 28.6 g/L and 90% of NB amount was about 13.5 g/L, that is half of 100% of MRS. The amount combined medium from the 55% of MRS and 90% of NB was about 42.0 g/L and was over half of 100% of MRS, which had 90% of the functional effect and yield. So the purpose of economic production can be achieved. The proportions of the appropriate medium can provide better nutrients and contribute to the promoted production and function of the exopolysaccharides (Huang et al., 2017; Inturri et al., 2017; Li et al., 2014; Tang et al., 2014; Wang et al., 2017). The reason might be that the EPS of both *Lactobacillus acidophilus* La and *Bifidobacterium adolescentis* Ba may have hydrophilic and lipophilic properties of biosurfactants (Chen et al., 2015). The characteristics of the hydrophilic and lipophilic properties of EPS can facilitate the compositions of antioxidants reacting with DPPH radicals (Chen et al., 2015). The backbone chain of EPS was composed of glucose, and galactose among others, which nutrients were provided by the combined medium (Huang et al., 2017). Thus, the combined medium promotes bacteria growth, EPS production, and antioxidant property. This suggests that it has the potential for industrial application as an economic prebiotic (Huang et al., 2017; Inturri et al., 2017). In summary, this study examined how to economically improve the productivity and yield of EPS and to efficiently reduce cost of EPS production. The EPS with optimal productivity and antioxidative ability was approached by the optimal production set of NB (90%) and MRS (55%) broth for the purpose of economic production. Therefore, the model of PPC might be economically compatible alternative to other methods used for improving the efficiency of production and antioxidative ability of EPS.

## CONFLICT OF INTEREST

The authors declare that there is no conflict of interest.

## ETHICAL STATEMENT

Human and animal testing is unnecessary in this study.
